# Different effects of *Atg2* and *Atg18* mutations on Atg8a and Atg9 trafficking during starvation in Drosophila^☆^

**DOI:** 10.1016/j.febslet.2013.12.012

**Published:** 2014-01-31

**Authors:** Péter Nagy, Krisztina Hegedűs, Karolina Pircs, Ágnes Varga, Gábor Juhász

**Affiliations:** Department of Anatomy, Cell and Developmental Biology, Eötvös Loránd University, Budapest H-1117, Hungary

**Keywords:** Atg, autophagy-related, PAS, phagophore assembly site, PI3P, phosphatidylinositol 3-phosphate, Ref(2)P, refractory to Sigma P, ULK, uncoordinated-51 like autophagy kinase, Vps, vacuolar protein sorting, WIPI, WD40 repeat domain phosphoinositide-interacting protein, Atg2, Atg7, Atg8a, Atg9, Atg18, Ref(2)P/p62

## Abstract

The Atg2–Atg18 complex acts in parallel to Atg8 and regulates Atg9 recycling from phagophore assembly site (PAS) during autophagy in yeast. Here we show that in Drosophila, both Atg9 and Atg18 are required for Atg8a puncta formation, unlike Atg2. Selective autophagic degradation of ubiquitinated proteins is mediated by Ref(2)P/p62. The transmembrane protein Atg9 accumulates on refractory to Sigma P (Ref(2)P) aggregates in *Atg7*, *Atg8a* and *Atg2* mutants. No accumulation of Atg9 is seen on Ref(2)P in cells lacking Atg18 or Vps34 lipid kinase function, while the Atg1 complex subunit FIP200 is recruited. The simultaneous interaction of Atg18 with both Atg9 and Ref(2)P raises the possibility that Atg18 may facilitate selective degradation of ubiquitinated protein aggregates by autophagy.

## Highlights

• Atg9 and Atg18 are required for autophagy upstream of Atg8a, unlike Atg2.
• Atg9 accumulates on Ref(2)P aggregates in *Atg8a*, *Atg7* and *Atg2* mutants.
• Ultrastructurally, Atg9 vesicles cluster around Ref(2)P aggregates in stalled PAS.
• Atg9 does not accumulate on Ref(2)P upon loss of Atg18 or Vps34, while FIP200 does.
• Atg18 simultaneously interacts with both Atg9 and Ref(2)P.

## Structured summary of protein interactions

**Ref(2)P**physically interacts with **Atg18** by anti tag coimmunoprecipitation (View interaction) **Atg18**physically interacts with **Atg2** by anti tag coimmunoprecipitation (View interaction) **CG8678**physically interacts with **Atg2** by anti tag coimmunoprecipitation (View interaction) **Atg18**physically interacts with **atg9** by anti tag coimmunoprecipitation (View interaction)

## 1 Introduction

Autophagy is a major catabolic pathway capable of degrading all kinds of intracellular material including proteins, lipids, polysaccharides, and nucleic acids. During its main pathway, phagophores capture cytosol and organelles to form autophagosomes, followed by the fusion of these double-membrane vesicles with lysosomes [1]. Autophagy was initially considered to be a non-specific, bulk degradation system, in contrast with the ubiquitin–proteasome pathway, in which individual polyubiquitinated proteins are recognized, unfolded and degraded in the inner proteolytic chamber of proteasomes. More recently, multiple studies showed that ubiquitination also signals for selective autophagic degradation, and characterization of ubiquitin-specific autophagy receptors revealed the molecular mechanism involved [2], [3]. Proteins such as p62 contain distinct domains mediating multimerization, ubiquitin binding and interaction with Atg8 family proteins. Atg8 is a ubiquitin-like protein bound to phagophores and autophagosomes through a lipid anchor [1], [4], [5]. Ubiquitinated proteins are captured into aggregates by binding to p62 multimers, and the interaction of p62 with Atg8-positive phagophores is considered to be responsible for their elimination by autophagy [2], [3], [6], [7], [8]. In contrast with this simple model, p62 was found to colocalize with proteins involved in the initiation of phagophores independent of the presence of mammalian Atg8 homologs such as LC3 [9]. These results suggest that additional factors also contribute to the recognition of p62 aggregates by phagophores.

Atg9 is the only transmembrane protein of core autophagy factors, and it likely supplies initial vesicles for phagophore nucleation from multiple membrane sources including endosomes, plasma membrane and Golgi [10], [11], [12]. Atg9 is considered to be an upstream factor in the hierarchy of autophagy-related (Atg) proteins in yeast, but the molecular determinants of Atg9 recruitment to the phagophore assembly site (PAS) are incompletely characterized [13]. A recent study shows that mammalian Atg9 is recruited to damaged mitochondria independent of lipidated Atg8 homologs and the upstream kinase Atg1/ULK1 during selective autophagic degradation of mitochondria [14]. In yeast, the Atg2–Atg18 protein complex is thought to act in parallel to the Atg8 system, and regulates Atg9 recycling from PAS [13], [15]. Diverse Atg18-like proteins are found in eukaryotes. The four mammalian homologs fall into two groups based on bioinformatic analysis: WIPI1/2 and WIPI3/4 [16], [17]. Of these, both WIPI2 and WIPI4 were suggested to promote autophagosome formation based on siRNA experiments in cultured cells [16], [18]. Yeast Atg18 and its paralog Atg21 belong to the WIPI1/2 group [16], [17]. Atg21 only functions in an autophagy-related biosynthetic pathway called cytoplasm to vacuole targeting (cvt) which delivers a subset of hydrolases to the vacuole under growth conditions, whereas Atg18 is required for both autophagy and cvt in yeast [19]. Ygr223c, the third Atg18 family protein in yeast, belongs to the WIPI3/4 group and regulates micronucleophagy, a selective autophagy pathway for degradation of nuclear components [16], [20].

Clear orthologs of most Atg proteins are found in the popular metazoan model Drosophila, and the p62 ortholog refractory to Sigma P (Ref(2)P) also promotes ubiquitinated protein aggregation [21], [22]. Single genes code for Atg9 and Atg2, and the WIPI1/2 homolog CG7986 is referred to as Atg18, as it is required for autophagy in Drosophila [23], [24], [25], [26]. The roles of another WIPI1/2 family Drosophila protein, CG8678, and of the WIPI3/4-like CG11975 are unknown [16], [17].

Here we show that loss of Atg2 or Atg18 have different consequences on Atg8a puncta formation and Atg9 recruitment to Ref(2)P aggregates during starvation in Drosophila, raising the possibility that these proteins act differently during autophagosome formation in Drosophila.

## 2 Materials and methods

### 2.1 Molecular cloning, immunoprecipitation and antibody production

Atg18 coding sequences were PCR amplified from LD38705 (DGRC), and cloned into appropriate vectors to generate R4-mCherry-Atg18, UAS-3xHA-Atg18, and UAS-3xFLAG-Atg18, respectively. Coding sequences were amplified from genomic DNA or GH07816 (DGRC) to generate UAS-3xFLAG-Atg2 or UAS-3xHA-CG8678, respectively. These UAS plasmids, together with UAS-3xHA-Atg9, UAS-3xFLAG-Ref(2)P and mt-Gal4, were used to transfect D.Mel-2 cells (Invitrogen), followed by processing for immunoprecipitation as described [25], [27]. His-tagged recombinant Atg9 protein fragment (amino acids 541–845) was purified from bacteria and used for immunization of rats as before [27].

### 2.2 Drosophila genetics

Flies were reared on standard cornmeal-yeast-agar diet, and well-fed mid-third instar larvae were floated in a 20% sucrose solution for 3 h in starvation experiments. Genotypes used in this study are RNAi lines *Atg9^GD10045^* (VDRC), *Atg9^HMS01246^*, *Atg9^JF02891^*, *Atg18^JF02898^*, *Atg8a^KK109654^*, *Atg2^JF02786^,* and *w^1118^* used as wild type (all from BDSC) [26], mutants *Atg7^d77^* [28], *Atg2^EP3697^/Df(3L)BSC119*, *Atg18^KG03090^/Df(3L)Exel6112*, *Atg8a^d4^* [23], [26], *cg-Gal4, UAS-Vps34^KD^* [29], and stocks for clonal analysis *hs-Flp; UAS-Dcr2; Act > CD2 > Gal4, UAS-GFPnls, R4-mCherry-Atg8a* and *hs-Flp; UAS-Lamp1-GFP; Act > CD2 > Gal4, UAS-Dcr2* [25], [26], [27]. *R4-mCherry-Atg18* transgenics were generated by Bestgene.

### 2.3 Western blots and histology

Western blots were carried out as described previously [25], [26], [27]. Dissected larval carcasses were processed for LysoTracker Red (Invitrogen) or immunostaining as before, using rabbit anti-Ref(2)P (1:2000), rat anti-Atg8a (1:300), rat anti-Atg9 (1:300), rat anti-FIP200 (1:300) and rat anti-mCherry (1:300) primary antibodies [25], [26], [27]. Preparations were photographed on an Axioimager M2 with Apotome2 (Zeiss), and original unmodified images were processed for statistical evaluation using ImageJ (NIH) and SPSS Statistics (IBM), as described [25], [26], [27]. Colocalizations were calculated either by the colocalization tool in ImageJ to obtain Mander’s colocalization coefficients for Atg8a-Ref(2)P, or by manual counting for Atg9-Ref(2)P. Immunogold labeling for ubiquitin was done as described [28]. Anti-Atg9 (1:30) and anti-Ref(2)P (1:60) double immunogold labeling was carried out on adult brains embedded into LR White resin (Sigma) to increase antigenicity, using secondary antibodies anti-rat conjugated to 10 nm gold (1:20, Sigma) and anti-rabbit conjugated to 18 nm gold particles (1:60, Jackson Immunoresearch).

## 3 Results

**Fig. 1:**
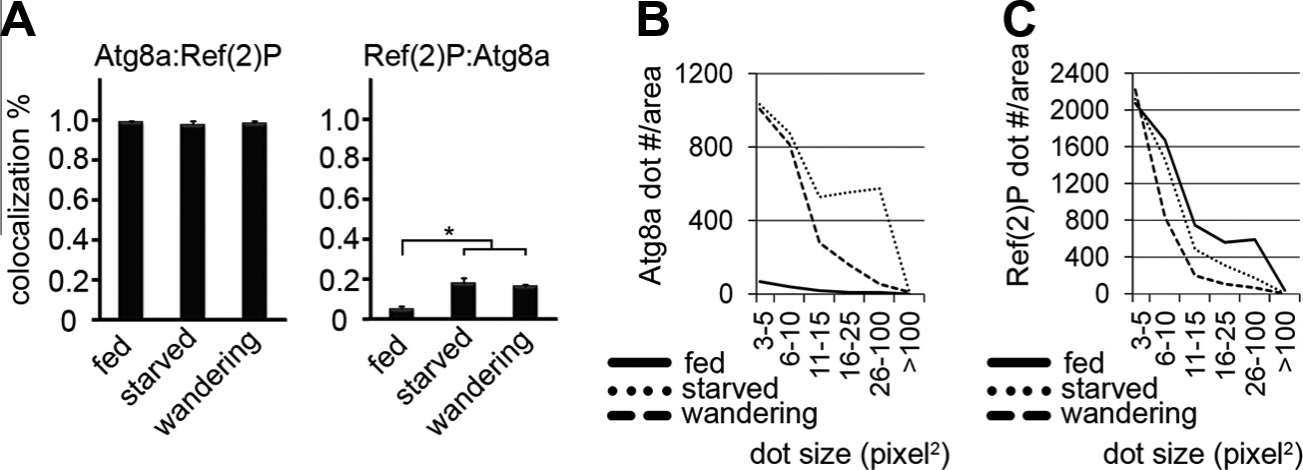
(A) Atg8a colocalizes with Ref(2)P in Drosophila fat body cells of fed, starved and wandering wild type L3 larvae. The colocalization of Ref(2)P with Atg8a increases during starvation or developmental autophagy, as much more autophagosomes are generated under these circumstances. *N* = 6–15 per stage, ^∗^*P* < 0.001, ANOVA, errors bars: S.D. (B) Atg8a-positive autophagosomes are induced by starvation or during the wandering stage. (C) Larger Ref(2)P aggregates observed in fat body cells of well-fed animals are eliminated during starvation or wandering.

**Supplementary Fig. S1:**
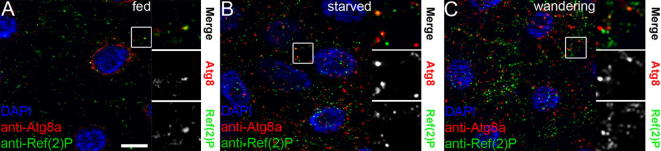
(A–C) Atg8a punctae colocalize with Ref(2)P dots in fat bodies of fed (A), starved (B) and wandering (C) animals. Bar in A equals 20 μm for A–C.

p62 aggregates are selectively captured into autophagosomes under both basal or starvation conditions in mammalian cells, as more than 90% of LC3-positive autophagosomes are also positive for p62 [6]. In line with that, most Atg8a-positive autophagosomes colocalized with Ref(2)P in fat bodies of well-fed, starved or wandering Drosophila larvae, respectively (Figs. 1A and S1). As expected, the number of Atg8a dots increased in response to autophagy induction (Fig. 1B). Strikingly, larger Ref(2)P aggregates seen under low autophagy level in fed cells disappeared during starvation-induced or developmental autophagy (Fig. 1C). Thus, Ref(2)P aggregates are selectively eliminated by autophagy in Drosophila.

**Fig. 2:**
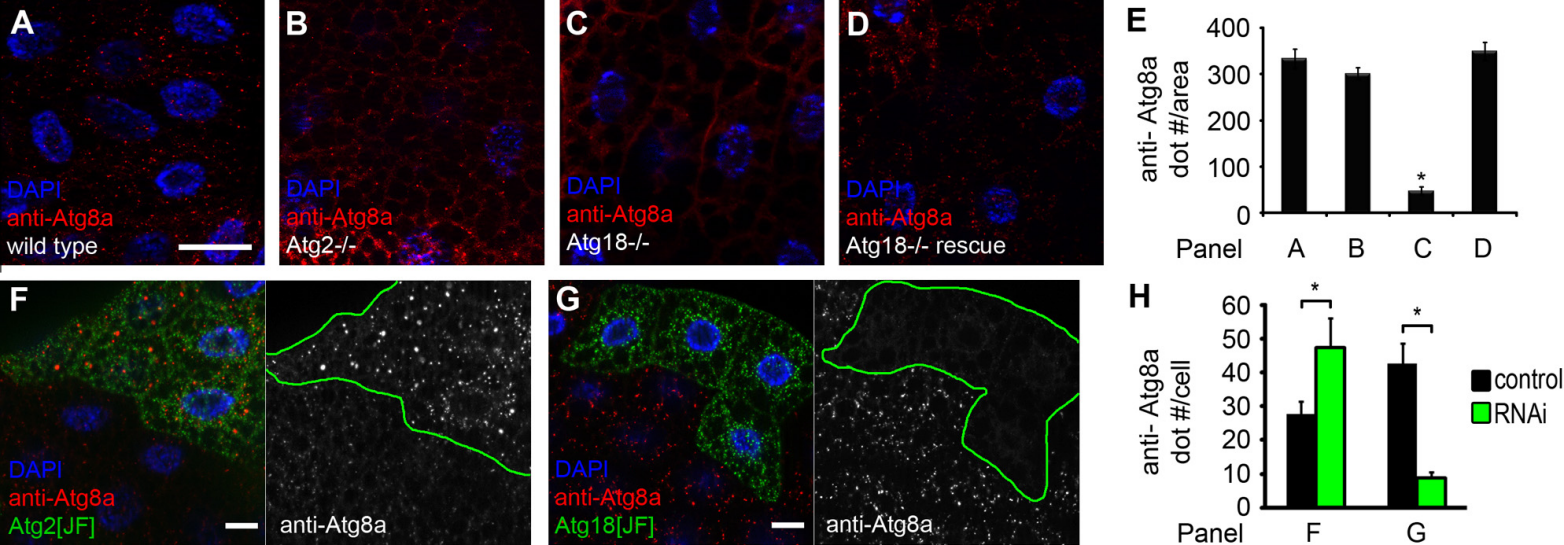
(A–D) Punctate endogenous Atg8a staining is seen in fat body cells of wild type (A) and *Atg2* mutant (B) starved larvae. The Atg8a dot formation defect of *Atg18* mutants (C) is rescued by transgenic expression of Atg18 (D). Note that Atg8a is pseudocolored red in panel D, as Alexa 488 was used to avoid bleedthrough from the mCherry channel. (E) Quantification of data shown in (A–D). *N* = 5/genotype, ^∗^*P* < 0.001, ANOVA, errors bars: S.D. (F) *Atg2* RNAi in GFP-positive cells does not block Atg8a puncta formation. Note that Atg8a structures in RNAi cells appear brighter, and a fraction of these are also bigger than Atg8a dots observed in surrounding control cells lacking GFP. (G) RNAi knockdown of *Atg18* in GFP-marked cell clones blocks Atg8a puncta formation. Note that the Atg8a dots that do form in RNAi cells appear smaller and are much less bright than those observed in neighboring control cells. (H) Quantification of data shown in F, G. *N* = 5/genotype, ^∗^*P* < 0.001, two-tailed, two-sample Student’s *T* test, errors bars: S.D. Bar in A equals 20 μm for A–D, F, G, and the relevant channels are shown in grayscale as indicated.

**Supplementary Fig. S2:**
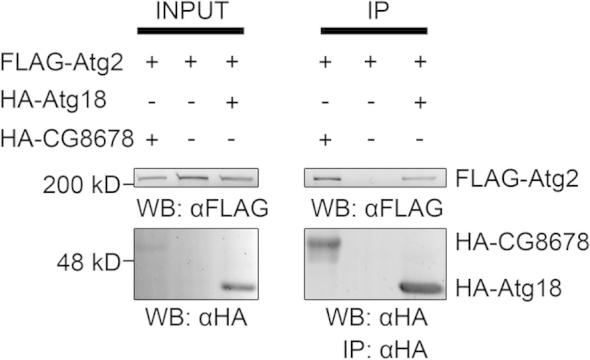
FLAG-Atg2 coprecipitates with both HA-Atg18 and HA-CG8678 in cultured Drosophila cells. Note that more Atg2 is coprecipitated with CG8678, even though the expression level of CG8678 is much lower than that of Atg18. IP: immunoprecipitation, WB: western blot.

Atg2 and Atg18 act in parallel to Atg8 in yeast, whereas Atg2 appears to function downstream of Atg8 family proteins in worms and mammals, respectively [18], [30]. We also found that punctate Atg8a structures form in fat bodies of starved *Atg2* mutants previously shown to be defective in autophagic degradation (Fig. 2A, B and E) [23], [26]. In contrast, Atg8a dots were rarely detected in starved *Atg18* mutants, and they were restored by expression of mCherry-Atg18 (Fig. 2C–E). Similarly, RNAi depletion of *Atg2* failed to block the formation of Atg8a-positive structures, while *Atg18* knockdown inhibited punctate Atg8a in GFP-marked RNAi cells (Fig. 2F–H). Immunoprecipitation experiments suggested that Atg2 may interact with Drosophila Atg18, and more efficiently with its paralog CG8678 (Fig. S2).

**Supplementary Fig. S3:**
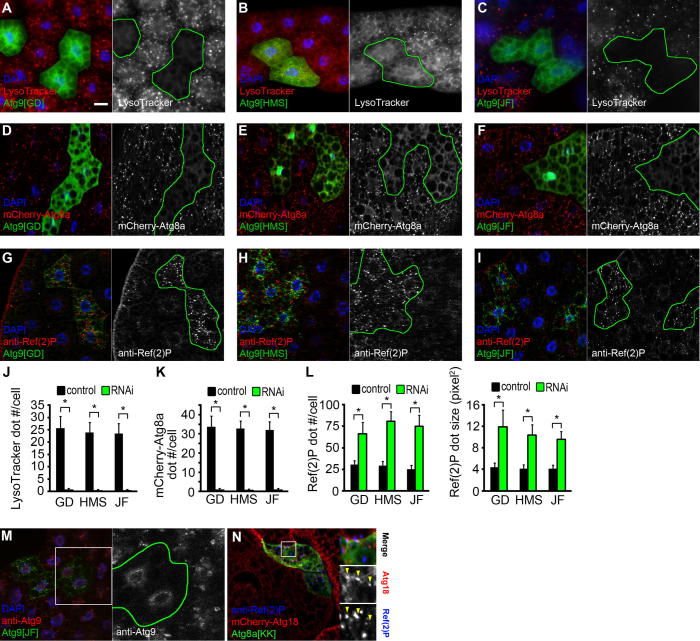
(A–I) Analysis of starvation-induced autophagy in genetic mosaics, where GFP-positive cells express RNAi transgenes to knock down *Atg9*, and surrounding non-GFP cells in the same tissue serve as an internal control. (A–C) Depletion of *Atg9* using either one of three independent RNAi transgenes blocks the generation of Lysotracker Red-positive autolysosomes. (D–F) *Atg9* knockdown mediated by these three independent transgenes prevents the formation mCherry-Atg8a-positive autophagosomes and autolysosomes. (G–I) The autophagic cargo Ref(2)P accumulates in cells with loss of *Atg9* function. (J–L) Quantification of data from A–C (J), D–F (K), G–I (L). *N* = 5–10/genotype, ^∗^*P* < 0.001, two-tailed, two-sample Student’s *T* test, errors bars: S.D. M: Our new polyclonal rat anti-Atg9 antisera specifically recognize punctate and clustered endogenous Atg9-positive structures in starved fat body, as these structures are missing from GFP-positive cells undergoing *Atg9* RNAi. N: mCherry-Atg18 overlaps with Ref(2)P in GFP-marked *Atg8a* RNAi cells. Bar in A equals 20 μm for A–I, M, N, and the relevant channels are shown in grayscale as indicated.

We have recently shown that Drosophila Atg9 is required for basal, Myc-induced or proteasome inactivation-induced autophagy, respectively [24], [25], [26]. Atg9 is also necessary for the starvation response, as expression of either one of three independent RNAi lines in GFP-marked cell clones prevented punctate LysoTracker staining, a commonly used marker of autolysosomes in the fat body (Fig. S3A–C and J). Moreover, formation of mCherry-Atg8a positive autophagosomes and autolysosomes were also blocked in knockdown cells, and the selective cargo Ref(2)P accumulated upon *Atg9* RNAi during starvation (Fig. S3D–I, K and L).

**Fig. 3:**
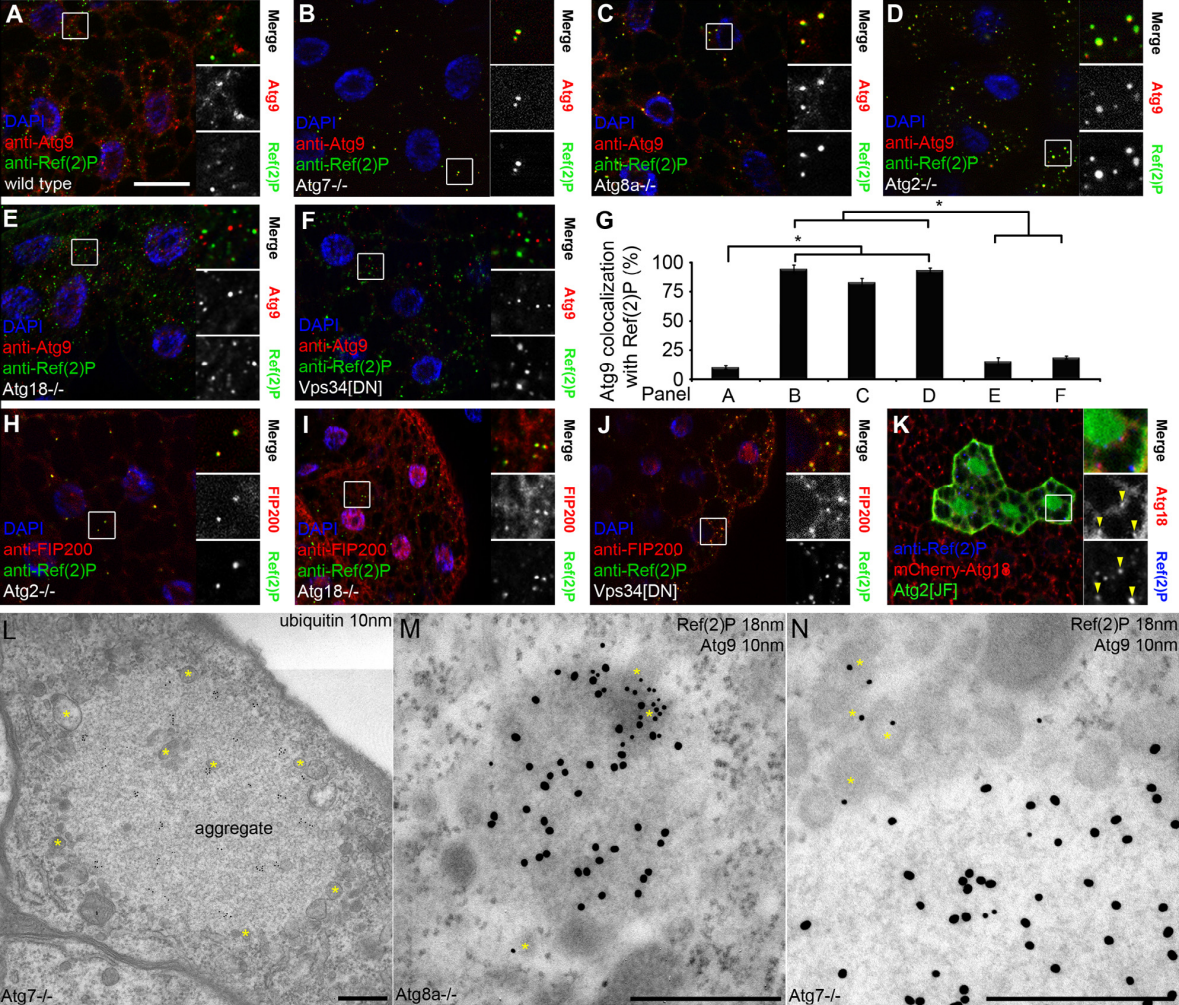
(A) Atg9 rarely colocalizes with Ref(2)P in wild type larvae. (B–D) A near-complete overlap of Atg9 and Ref(2)P is seen in *Atg7* (B), *Atg8a* (C) and *Atg2* (D) mutants. (E, F) Atg9 and Ref(2)P rarely colocalize in *Atg18* mutants (E) or in fat bodies expressing dominant-negative Vps34 (F). (G) Quantification of data from A–F. *N* = 5/genotype, ^∗^*P* < 0.001, ANOVA, errors bars: S.D. (H–J) FIP200 accumulates on Ref(2)P aggregates in starved *Atg2* (H) and *Atg18* mutants (I), and in larvae expressing dominant-negative Vps34 in fat bodies (J). (K) mCherry-Atg18 colocalizes with Ref(2)P in GFP-marked *Atg2* RNAi cells. (L) Immunogold labeling shows accumulation of numerous vesicles (some of which are indicated by asterisks) around and occasionally also inside aggregates containing ubiquitinated proteins in *Atg7* mutant neurons. (M, N) Double immunogold labeling shows endogenous Atg9-positive vesicles (asterisks) accumulating at the periphery of Ref(2)P aggregates in *Atg8a* (M) and *Atg7* (N) mutant neurons. Bar in A equals 20 μm for A–F, H–K, and boxed areas are shown enlarged as indicated. Bars equal 300 nm in L–N.

We raised polyclonal antibodies against Drosophila Atg9, which specifically recognized Atg9 dots and clusters in starved fat body cells (Fig. S3M). Phagophores assemble at aggregates of the selective cargo pro-aminopeptidase I in yeast, and p62-positive aggregates may play similar roles in metazoans [3]. In wild type cells, the overlap of Atg9 with Ref(2)P was low (Fig. 3A and G), in line with the transient localization of Atg9 to forming phagophores described in mammalian cells [10], [31]. Loss of downstream Atg genes results in the accumulation of upstream factors in stalled PAS, both in yeast and mammals [13], [32]. We therefore carried out similar analyses for Atg9 and Ref(2)P. Nearly all Ref(2)P aggregates colocalized with Atg9 in null mutants of *Atg7* encoding the E1-like enzyme required for Atg8a lipidation and membrane association, and in *Atg8a* nulls (Fig. 3B, C and G).

Aggregates of Ref(2)P also overlapped with Atg9 in *Atg2* mutant fat cells in starved fly larvae (Fig. 3D and G), consistent with the role of Atg2 downstream of (or parallel to) Atg8a. Strikingly, colocalization of these two proteins was low in *Atg18* mutants (Fig. 3E and G). Atg18 is a phosphatidylinositol 3-phosphate (PI3P) effector [33], which prompted us to test whether Vps34, the lipid kinase producing this phospholipid, is involved in Atg9 recruitment to Ref(2)P. Colocalization of Atg9 with Ref(2)P was again low in fat bodies expressing dominant-negative Vps34 (Fig. 3F and G). These data suggested that either Atg9 or Ref(2)P is not recruited to the PAS in the absence of Atg18 or Vps34 function. To distinguish between these possibilities, we looked at the localization of FIP200/Atg17. FIP200 is a subunit of the Atg1 kinase complex, and it accumulates on Ref(2)P aggregates in fat body cells of starved *Atg7* null mutant larvae [34]. FIP200 was also enriched on Ref(2)P aggregates in cells lacking Atg2, Atg18 or Vps34 function (Fig. 3H–J), suggesting that Atg9 recruitment to PAS may specifically be affected by the absence of Atg18 or Vps34. Moreover, overlapping mCherry-Atg18 and Ref(2)P structures were readily observed in *Atg2* and *Atg8a* RNAi cells (Figs. 3K and S3N).

These data suggested that Ref(2)P aggregates with associated upstream Atg proteins may represent stalled PAS in *Atg* mutants. The ultrastructure of protein inclusions is well-characterized in *Atg* mutant neurons of adult flies and mice, respectively [28], [35], [36]. These aggregates contain ubiquitinated protein, and are often surrounded by small vesicles (Fig. 3L). Double immunolabeling experiments revealed that at least a subset of vesicles associated with Ref(2)P-positive inclusions are positive for endogenous Atg9 in neurons of *Atg7* and *Atg8a* null mutant adult brains (Fig. 3M and N).

**Fig. 4:**
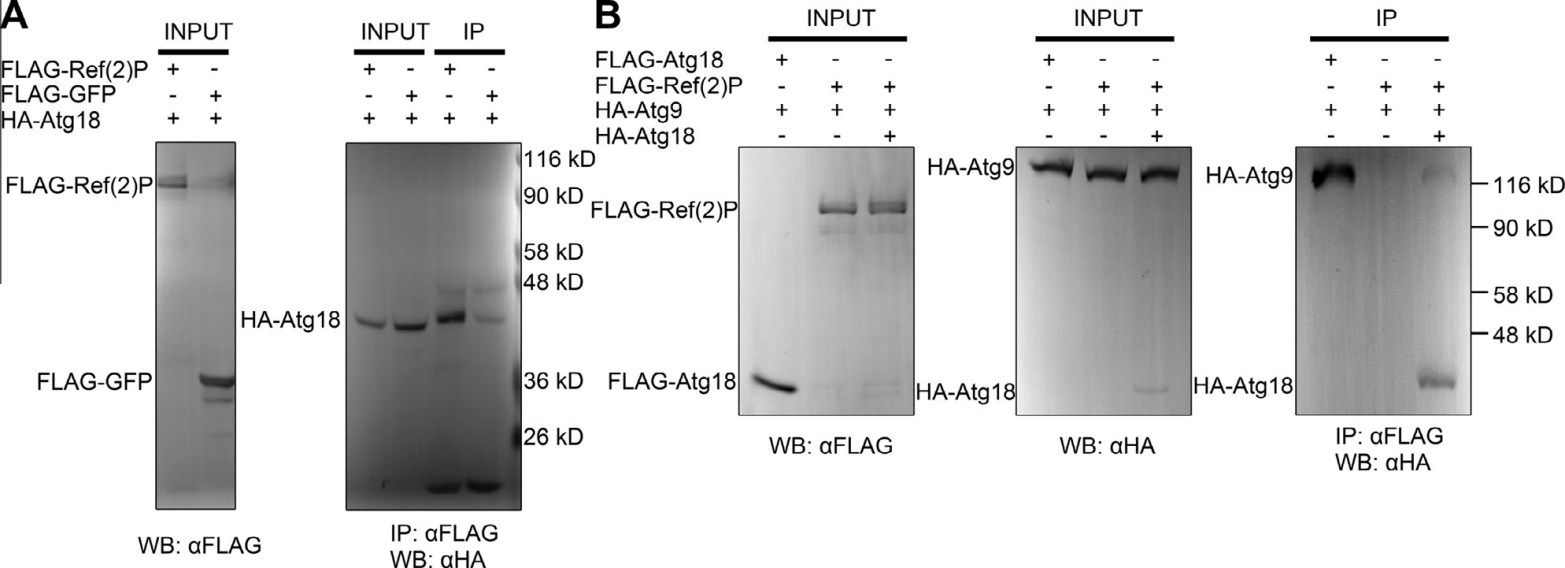
(A, B) Analysis of protein interactions in cultured cells. (A) HA-Atg18 coprecipitates FLAG-Ref(2)P. FLAG-GFP is shown as negative control. (B) HA-Atg9 coprecipitates with FLAG-Atg18, but not with FLAG-Ref(2)P. Coexpression of HA-Atg18 results in coprecipitation of HA-Atg9 with FLAG-Ref(2)P. IP: immunoprecipitation, WB: western blot.

The lack of Atg9 accumulation on Ref(2)P aggregates in *Atg18* mutants raised the possibility that Atg18 may facilitate Atg9 recruitment to Ref(2)P. Indeed, HA-Atg18 coprecipitated with FLAG-Ref(2)P in cultured cells (Fig. 4A). HA-Atg9 showed strong binding to FLAG-Atg18, but not to FLAG-Ref(2)P (Fig. 4B), suggesting that the colocalization of Atg9 with Ref(2)P may not map a direct physical interaction. We hypothesized that Atg18 may potentially facilitate Atg9 recruitment to Ref(2)P aggregates by simultaneously binding to both proteins. In line with this model, coexpression of HA-Atg18 resulted in coprecipitation of HA-Atg9 with FLAG-Ref(2)P (Fig. 4B).

## 4 Discussion

Previous studies established the importance of the interaction between p62 and Atg8 family proteins (such as LC3) during selective autophagy in mammalian cells [2], [3], [6]. A recent live imaging-based report shows that these two proteins are recruited together to the PAS, but later than the Atg1/ULK kinase complex, the Vps34 lipid kinase complex and its effectors such as WIPI1 [31]. How p62 and LC3 are recruited to the PAS is still not understood completely. Several interactions have been recently described between Atg proteins belonging to distinct functional groups and complexes, which likely cooperately coordinate the recruitment of downstream factors. For instance, LC3-interacting regions have been characterized in ULK1/Atg1 and its binding partners Atg13 and FIP200, which may facilitate LC3 recruitment to PAS [37].

Atg9 likely supplies lipids in the form of small vesicles to initiate phagophore formation, and most of it is normally recycled from phagophores [10], [31]. Atg18 is a phospholipid effector, and its complex with Atg2 regulates Atg9 recycling from phagophores in yeast [15]. Atg18 and Atg2 are considered to function together, genetically in parallel to Atg8 in yeast [13]. A recent paper shows that Atg18 recruitment to PAS requires PI3P and Atg2, and the Atg18-related protein Atg21 also appears to facilitate its localization to pro-aminopeptidase I aggregates in yeast [38]. Loss of Atg2 leads to formation of stalled phagophores in worms and mammalian cells, and it does not block the recruitment of worm Atg18 to protein aggregates [18], [30], which are entirely consistent with our findings in Drosophila. Loss of Atg18 suppressed Atg8a puncta formation unlike the lack of Atg2 function, indicating that Atg18 acts upstream of Atg8a in Drosophila. Similar to our findings, RNAi depletion of the *Atg18* homolog *WIPI2* was found to prevent LC3 puncta formation in cultured mammalian cells, and mutation of *Atg18* attenuates efficient Atg8 puncta formation in the 50 cell stage worm embryo [16], [18]. Interestingly, Atg18 and Atg21 were also suggested to promote the recruitment of lipidated Atg8 to the PAS in yeast, based on experiments carried out in a multiple knockout strain which contains only a subset of *Atg* genes [19].

Our data raise the possibility that the selective autophagy cargo Ref(2)P may be used as a potential PAS marker in *Atg* mutant Drosophila cells, as most Ref(2)P aggregates become positive for upstream Atg proteins. The Atg1 complex subunit FIP200 is enriched on Ref(2)P aggregates in the absence of Atg7, Atg2, Atg18 or Vps34 function. Atg9 also accumulates on Ref(2)P aggregates in *Atg7*, *Atg8a* and *Atg2* mutants, but not in *Atg18* mutants. Atg18 family proteins contain a WD40 domain with 7 beta-propellers. This structure likely enables the binding of Atg18 to multiple targets simultaneously, including the autophagy proteins Atg2 and Atg9 that we also show here, and PI3P on autophagic membranes [15], [19], [33], [38]. In line with the interaction of Atg18 with this phospholipid, accumulation of Atg9 on Ref(2)P aggregates also requires the lipid kinase Vps34. Our data suggest that Atg18 may play an important role in phagophore nucleation, potentially by facilitating the recruitment of Atg9-containing vesicles to Ref(2)P for selective capture and degradation of ubiquitinated protein aggregates through autophagy in Drosophila.
